# A Relationship between the Structures and Neurotoxic Effects of Aβ Oligomers Stabilized by Different Metal Ions

**DOI:** 10.1021/acschemneuro.3c00718

**Published:** 2024-02-28

**Authors:** Sean Chia, Rodrigo Lessa Cataldi, Francesco Simone Ruggeri, Ryan Limbocker, Itzel Condado-Morales, Katarina Pisani, Andrea Possenti, Sara Linse, Tuomas P. J. Knowles, Johnny Habchi, Benedetta Mannini, Michele Vendruscolo

**Affiliations:** † Centre for Misfolding Diseases, Yusuf Hamied Department of Chemistry, University of Cambridge, Cambridge CB2 1EW, U.K.; ‡ Department of Biochemistry & Structural Biology, Center for Molecular Protein Science, Lund University, PO box 124, 221 00 Lund, Sweden; § Department of Physics, Cavendish Laboratory, Cambridge CB3 0HE, U.K.

**Keywords:** Alzheimer’s disease, metal ions, amyloid-β peptide, protein misfolding, protein aggregation, protein oligomers

## Abstract

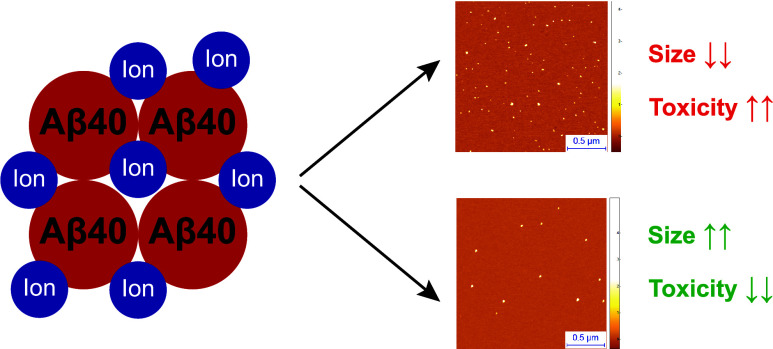

Oligomeric assemblies of the amyloid β peptide (Aβ) have been investigated for over two decades as possible neurotoxic agents in Alzheimer’s disease. However, due to their heterogeneous and transient nature, it is not yet fully established which of the structural features of these oligomers may generate cellular damage. Here, we study distinct oligomer species formed by Aβ40 (the 40-residue form of Aβ) in the presence of four different metal ions (Al^3+^, Cu^2+^, Fe^2+^, and Zn^2+^) and show that they differ in their structure and toxicity in human neuroblastoma cells. We then describe a correlation between the size of the oligomers and their neurotoxic activity, which provides a type of structure–toxicity relationship for these Aβ40 oligomer species. These results provide insight into the possible role of metal ions in Alzheimer’s disease by the stabilization of Aβ oligomers.

## Introduction

Alzheimer’s disease (AD) is the most common cause of dementia.^1^ At the molecular level, AD has been associated, along with over 50 other disorders, with the misfolding and aggregation of normally monomeric peptides and proteins into amyloid deposits.^2,3^ It is also increasingly apparent that the complexity of the aggregation process can lead to the formation of a wide variety of aggregated structures, which exert different cytotoxicities.^4−7^ In particular, many recent studies have focused on diffusible, transient oligomers formed during the aggregation process and their related neurotoxic behaviors.^8−12^ Certain structural elements have been postulated to affect their ability to cause cellular dysfunction. It has been shown, for example, that oligomers which are smaller, and with greater exposure of hydrophobic patches, are generally more cytotoxic.^5,6,9,13,14^ In addition to the intrinsic propensity to form polymorphic aggregated structures, external factors can also strongly influence the formation of different types of oligomers, such as the complex and crowded cellular environment with a multitude of molecules, proteins, and lipid membranes.^15−17^

In the case of AD, although the deposition of Aβ into amyloid plaques is a molecular signature of the disease,^8^ the primary species leading to cellular dysfunction may be oligomeric assemblies that are precursors of the mature amyloid state.^15,16,18^ A relevant environmental condition to consider in this context is the presence of metal ions, which are strongly associated with the pathology of AD.^19−22^ In particular, some metal ions that exist at high total concentration in the brain, such as zinc, copper, and iron ions, have been observed to directly interact with the aggregation of Aβ, and for the case of zinc ions, inhibit the elongation of Aβ fibrils, and stabilize cytotoxic oligomers.^10,23−26^ In fact, considering the strong association of these metal ions with the pathogenesis of AD, recent therapeutic efforts have been pursued in targeting these metal ion levels via supplementation or chelation therapies.^25,27,28^

In this work, we characterize the molecular mechanisms of action of four different metal ions, namely, Zn^2+^, Cu^2+^, Al^3+^, and Fe^2+^, on the aggregation of the most abundant form of Aβ40 (the 40-residue form of Aβ).^29^ We first observe that distinct oligomers are formed in the presence of these different metal ions, which differ in their size distribution. Through a range of biophysical techniques, we also show that these oligomers possess different physicochemical properties, such as the extent of exposed hydrophobic surface and β-sheet structure. Further, we find that these oligomers induce different levels of cellular dysfunction to human neuroblastoma cells, such as the level of reactive oxygen species (ROS) production and Ca^2+^ influx. Based on these findings, we reveal a correlation between the size of the oligomers and their ability to induce cellular dysfunction. These results suggest a possible role of metal ions in AD by the stabilization of Aβ40 oligomers and identify structural determinants of the cellular toxicity of these assemblies.

## Results

### Stabilization of Aβ40 Oligomers by Different Metal Ions

**Figure 1:**
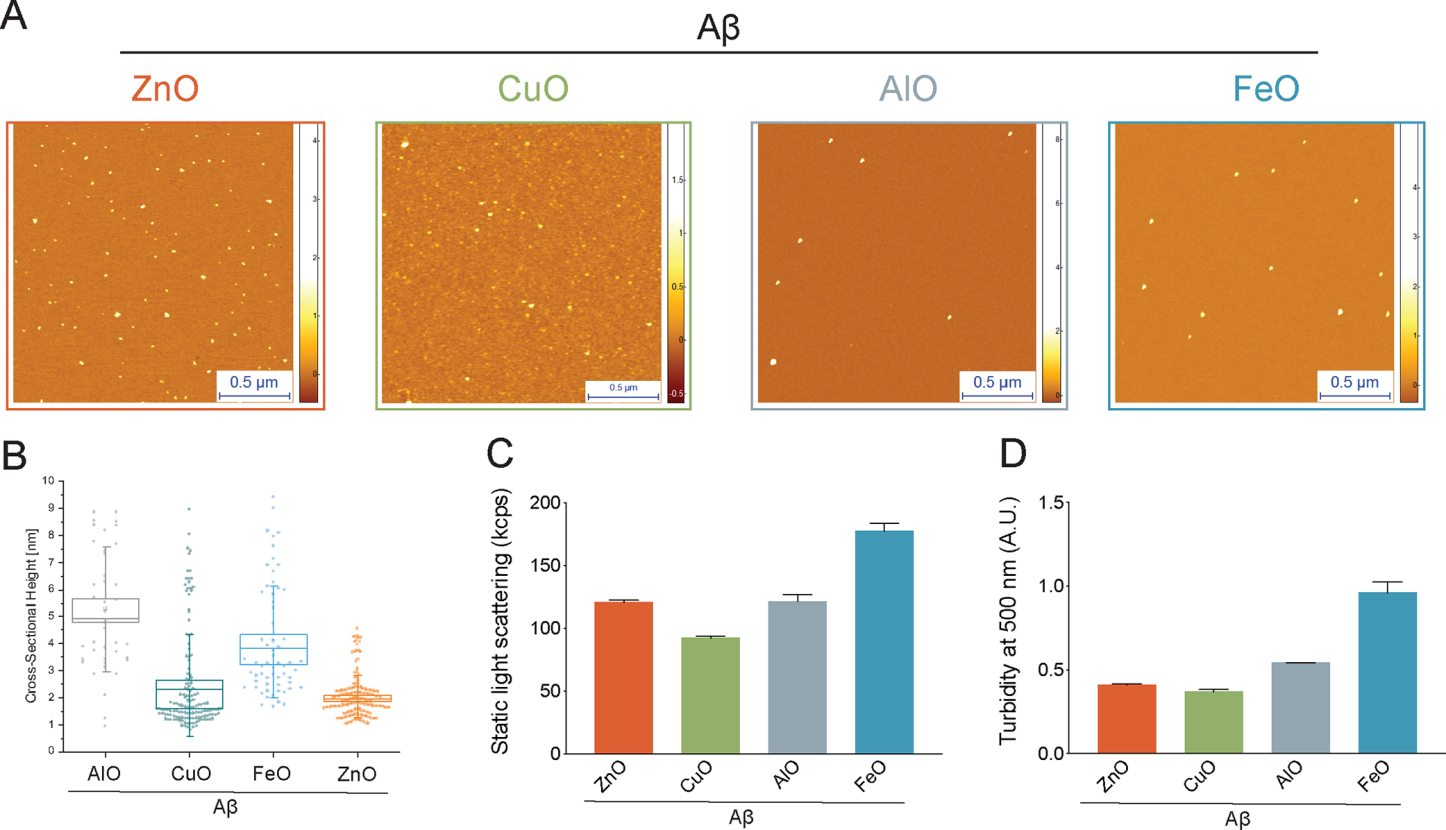
Comparison of the morphology and size distribution of Aβ40 oligomers stabilized by different metal ions (Zn^2+^, Cu^2+^, Al^3+^, Fe^2+^). (A) AFM morphology maps of Aβ-ZnO (orange), Aβ-CuO (green), Aβ-AlO (gray), and Aβ-FeO (blue). (B) Statistical analysis of the height distributions of the different Aβ40 oligomers shown in (A). (C, D) Measurements of static light scattering (C) and turbidity at 500 nm (D) for the Aβ40 oligomers. Error bars represent the s.e.m. (*N* = 2).

Zn^2+^ has previously been shown to interact with Aβ40 by redirecting the aggregation process into a higher prevalence of oligomeric species.^10^ Previous time course studies had determined that for a specific protocol of preparation, the presence of Zn^2+^ ions causes the formation of kinetically trapped stable Aβ40 oligomers that otherwise convert more rapidly to mature Aβ40 fibrils in its absence.^10^ Here, we adapted the same protocol based on the use of organic solvents, incubation in buffer with cosolvent, sonication, and isolation by centrifugation to generate kinetically stable Aβ40 oligomers, this time in the presence of 1:10 protein to four different metal ions, namely, Zn^2+^(Aβ-ZnO), Cu^2+^(Aβ-CuO), Al^3+^(Aβ-AlO), and Fe^2+^(Aβ-FeO) (see the Materials and Methods section). To characterize the aggregates formed in the presence of these ions, we used atomic force microscopy (AFM) to acquire high-resolution three-dimensional (3D) morphology maps of these structures (Figure 1A). From the acquired maps, we could observe spheroidal aggregates formed in the presence of the different metal ions, which is considered as one of the hallmark morphological features of oligomers.^6^ Upon analysis of the height distributions of the oligomers formed in the presence of the different ions, we observed different ion-dependent size distributions (Figure 1B). Specifically, Aβ-ZnO and Aβ-CuO displayed a smaller size distribution of approximately 2–2.5 nm, while Aβ-AlO and Aβ-FeO had a bigger size distribution centered at approximately 5 and 4 nm, respectively. As orthogonal approaches in measuring the size of the different oligomeric aggregates, we also employed static light scattering and turbidimetry (Figure 1C,D). As we observed by AFM, the average scattering count and turbidity measured showed the general sizes of aggregates of Aβ-AlO and Aβ-FeO to be bigger than Aβ-ZnO and Aβ-CuO. These measurements also suggest that Aβ-FeO aggregates are bigger than Aβ-AlO, unlike that observed from the AFM data. From the AFM data, a wider, more heterogeneous size distributions of Aβ-AlO and Aβ-FeO were observed as compared to Aβ-ZnO and Aβ-CuO, which could account for the disparity observed between the bulk and single molecule measurements (Figure 1), and from the fact that AFM requires adhesion to the surface for an oligomer to be observable. Further, to determine the role of the 1:10 protein:metal stoichiometry used for the formation of these kinetically stable oligomers, we studied the aggregation process of Aβ40 in the presence of increasing concentrations of the four different metal ions (Figure S1). We observed that the aggregation of Aβ40 was significantly affected in the presence of Zn^2+^, Cu^2+^, Al^3+^, and Fe^2+^ ions. However, depending on the identity of the metal ion, the molar equivalents of ions required for complete suppression of the Aβ40 aggregation process differed. For instance, a 0.25 mol equiv of Zn^2+^ ions was required to inhibit the aggregation process of Aβ40, while a 10 mol equiv of Fe^2+^ ions was required for a similar effect (Figure S1). Indeed, the 1:10 protein/metal stoichiometry used in the generation of the oligomers was also observed to be the amount required for all metal ions to significantly inhibit the aggregation process of Aβ40. Finally, we performed immune-diffusion sizing (IDS) of Aβ-ZnO and Aβ-FeO to measure the hydrodynamic radius of these stabilized oligomers (Figure S2). We found an *R*_H_ of 1.3 ± 0.1 and 1.8 ± 0.4 nm, respectively, in agreement with the values as derived from the AFM data, further confirming that oligomers of different sizes can form in the presence of the different ions (Figure S2).

### Aβ Oligomers Stabilized by Different Ions Possess Distinct Structural Properties

**Figure 2:**
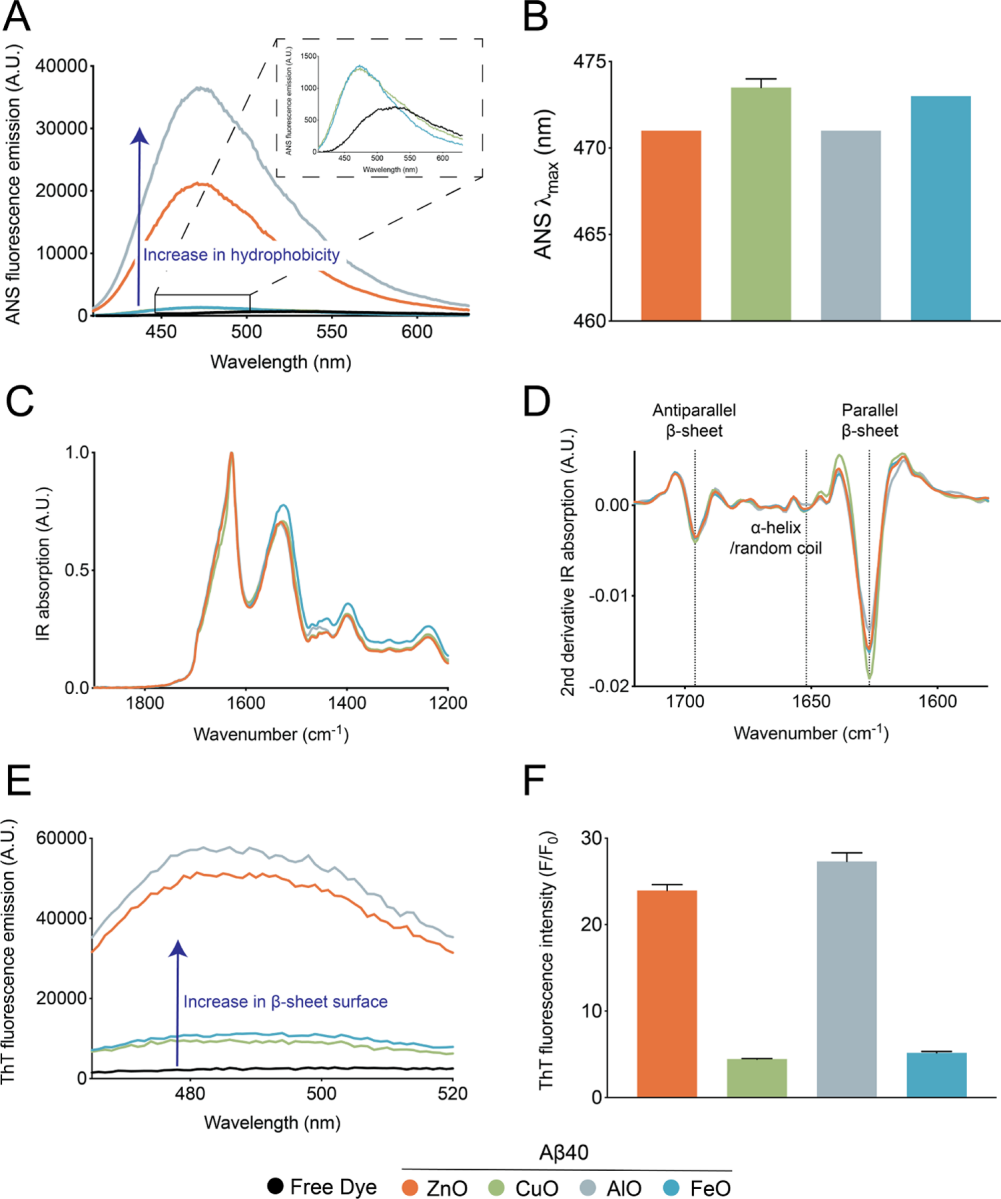
Structural properties of the four Aβ40 oligomer species studied in this work. (A) ANS fluorescence spectra of Aβ-ZnO (orange), Aβ-CuO (green), Aβ-AlO (gray), and Aβ-FeO (blue). The inset shows a magnified portion of the ANS spectra, with the free dye (black), Aβ-CuO, and Aβ-FeO. (B) Wavelength of the maximum ANS emission fluorescence (λ_max_) of the spectra in (A). (C, D) ATR-FTIR (C) and 2nd derivative ATR-FTIR (D) of Aβ-ZnO, Aβ-CuO, Aβ-AlO, and Aβ-FeO. All Aβ oligomers display parallel and antiparallel β-sheet structure. (E) ThT fluorescence spectra of Aβ-ZnO, Aβ-CuO, Aβ-AlO, and Aβ-FeO. (F) *F*/*F*_0_ ratio between the ThT fluorescence at 480 nm in the presence (*F*) and absence (*F*_0_) of Aβ oligomers as obtained from the spectra in (E). Error bars represent the s.e.m. (*N* = 2).

Certain structural properties of oligomers have been shown to modulate their neurotoxic activity.^9,14,15,34^ To investigate these links in the case of the Aβ oligomers studied in this work, we used a range of biophysical approaches in order to assess their structural characteristics. We first sought to measure the degree of hydrophobic surface exposure of the oligomers by means of the fluorescent probe 8-anilinonaphthalene-1-sulfonate (ANS) (Figure 2A,B). Upon binding to a hydrophobic patch, the fluorescence emission intensity of ANS increases, accompanied by a blue shift in its maximum emission wavelength (λ_max_).^30^ When incubated with the four different types of oligomers, we observed an increase in the ANS fluorescence emission compared to the buffer, suggesting the presence of exposed hydrophobic surfaces in the oligomer structures (Figure 2A). We also observed different intensities of ANS fluorescence emission depending on the type of oligomer. In particular, the fluorescence emission gain caused by Aβ-CuO and Aβ-FeO was lower than that caused by Aβ-ZnO, and Aβ-AlO, which exhibited the highest fluorescence emission intensity. The relatively stronger emission signal in the fluorescence emission was also accompanied by a greater wavelength shift of the emission maximum, suggesting that the oligomeric species forming in the presence of Al and Zn possess, on average, a higher degree of hydrophobic surface exposure than those formed with Cu and Fe (Figure 2B).

Besides assessing the hydrophobic surface exposure, we also measured the secondary structure of the oligomers by means of attenuated total reflection Fourier transform infrared (ATR-FTIR) spectroscopy (Figure 2C,D). Specifically, by assessing the position and the shape of the amide band I, we determined the secondary and quaternary structural organization of the oligomers.^31^ We observed that all of the oligomers contained strong bands at approximately 1628 cm^–1^, suggesting the presence of intermolecular parallel β-sheet structure (Figure 2D). Further, we also observed a significant band corresponding to approximately 1696 cm^–1^, which is indicative of the antiparallel β-sheet structure. Finally, a very weak signal between 1630 and 1660 cm^–1^ corresponding to α-helical and random coil conformations was also observed in all four oligomer species (Figure 2D). From these results, we observed that the overall secondary structure is conserved in all four oligomeric species tested here and consisted of mostly parallel and antiparallel β-sheet structure, without any significant difference in the ATR-FTIR spectra (Figure 2C,D). Considering the extensive β-sheet structure detected by ATR-FTIR, we further probed this property by means of the fluorescent probe thioflavin T (ThT), whose fluorescence emission increases dramatically upon binding to β-sheets of amyloid structures^32^ (Figure 2E,F). While a significant increase in the ThT fluorescence emission signal was observed for all four types of oligomers, suggesting that they may be fibrillar oligomers, the extent of emission gain was different between the distinct types of oligomers (Figure 2E,F). In particular, the ThT fluorescence emission was higher in the cases of Aβ-ZnO and Aβ-AlO as compared to Aβ-CuO and Aβ-FeO. These results suggest that although no significant difference in the overall secondary structure of the oligomers (as observed by ATR-FTIR) could be observed, the higher quantum yield of ThT binding of Aβ-ZnO and Aβ-AlO as compared to Aβ-CuO and Aβ-FeO implies a difference in surface character between the two classes of oligomers.

Subsequently, we probed the epitopes of these oligomers by dot blot assays using the conformation specific antibodies OC and A11, as well as the 6E10 antibody that is specific for the N-terminus of Aβ (Figure S3). From the dot blots, we observed that all four types of oligomers were reactive to the OC and 6E10 antibodies but did not show any significant reactivity to the A11 antibody (Figure S3). It is thus likely that the four types of oligomers generated resemble OC-reactive fibrillar oligomers in previous reports, which are structurally distinct from the prefibrillar A11-reactive oligomers.^33^ All in all, our results suggest an overall parallel and antiparallel β-sheet secondary structure across the different oligomers, resembling fibrillar oligomers. However, there appear to be also specific differences in their surface properties, such as their hydrophobic and β-sheet surfaces. This appears to be independent of their size differences, e.g., Aβ-ZnO and Aβ-CuO are structurally different despite similar sizes, and likewise for Aβ-AlO and Aβ-FeO.

Finally, we also sought to test the stability of these oligomers, particularly if there was any disassembly to monomers or further conversion to fibrils over time. First, we performed a time course assay by incubating the oligomers at 37 °C and monitoring their ThT binding properties over time (Figure S4). We observed no significant increase in the ThT fluorescence intensity in any Aβ-O, suggesting that the oligomers did not substantially assemble further into fibrils or disassemble into monomers. We further probed their stability properties by studying the aggregation process of Aβ40 monomers in the presence of increasing concentrations of each Aβ-O (Figure S5). In the scenario of disassembly, the increased concentrations of monomers (disassembled from oligomers) spiked into the solution would accelerate the aggregation process due to the greater pool of monomers available for self-assembly reactions. Similarly, in the other scenario of further assembly into fibrils, the increased concentrations of fibrils (further assembled from oligomers) spiked into the solution would seed the formation of new aggregates, and accelerate the aggregation process in general.^10^ Interestingly, we observed that Aβ-O delayed the aggregation process instead, albeit to different extents depending on the type of oligomer (Figure S5). In particular, Aβ-ZnO appeared to exert much more significant inhibitory potency, while Aβ-FeO appeared to exert the least significant inhibition. This suggests that Aβ-O may have the ability to trap and sequester Aβ40 monomers and prevent their conversion to fibrils.^10^ The overall lack of seeding and change in ThT fluorescence suggest that all Aβ-O complexes remain relatively stable and do not convert easily to other structural conformations over time.

### Aβ Oligomers Stabilized by Different Ions Exhibit Different Cellular Toxicity Levels

**Figure 3:**
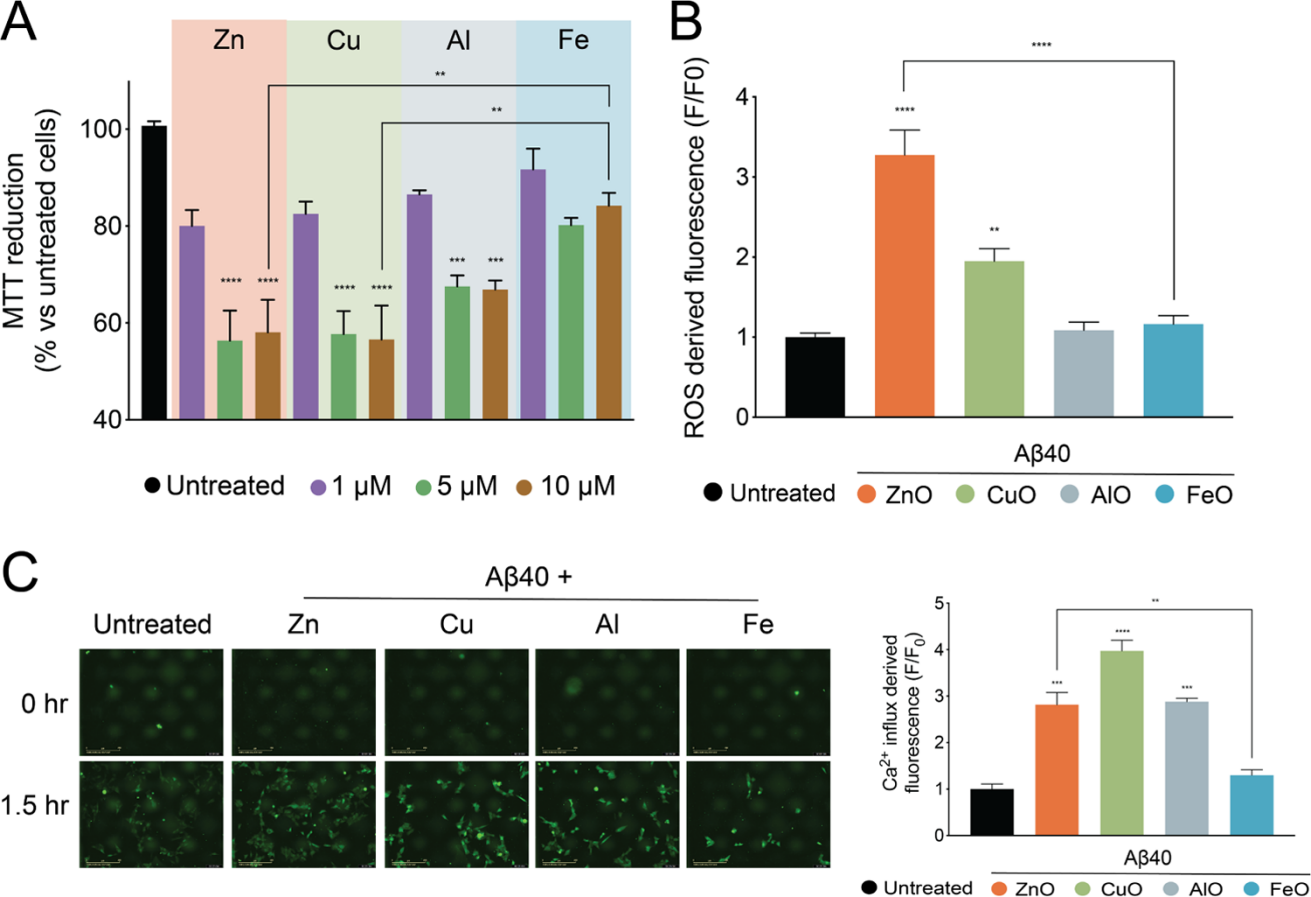
Aβ40 oligomers stabilized by different metal ions exert diverse cytotoxic activities in human neuroblastoma cells. (A) Cell viability determined by MTT reduction in the presence of 1 μM (purple), 5 μM (green), and 10 μM (brown) of Aβ-ZnO, Aβ-CuO, Aβ-AlO, or Aβ-FeO. (B) *F*/*F*_0_ ratio between the ROS-derived fluorescence intensity in the absence (*F*_0_) and in the presence (*F*) of 5 μM of each Aβ-O (represented in different colors), taken after 4 h following the treatment of the cells with the oligomers. (C) Representative images indicating fluorescence of the Fluo-4AM dye before and after 1.5 h treatment of the cells with 5 μM of each Aβ-O. (D) *F*/*F*_0_ ratio between the Ca^2+^ influx-derived fluorescence intensity in the absence (*F*_0_) and in the presence (*F*) of 10 μM of each Aβ-O, taken after 1.5 h following the treatment of the cells with the oligomers. Error bars represent the s.e.m. (*N* = 3 biological replicates for (A), *N* = 6 for (B), *N* = 3 for (C)). * *p* ≤ 0.05, ** *p* ≤ 0.01, *** *p* ≤ 0.001, **** *p* ≤ 0.0001 relative to untreated cells are shown.

The structural properties of an oligomer have been shown to influence its ability to disrupt cellular function.^34^ In light of their different structural properties, we sought to assess the cytotoxicities of the oligomers in human neuroblastoma SH-SY5Y cells. First, we assessed cellular dysfunction in the presence of the oligomers using the 3-(4,5-dimethylthiazol-2-yl)-2,5-diphenyltetrazolium bromide (MTT) test, where the viability of healthy cells is monitored through their mitochondrial ability to reduce the MTT molecule^35^ (Figures 3A and S6). In the presence of 5 and 10 μM oligomers, we observed a reduction in cell viability in the cases of Aβ-ZnO, Aβ-CuO, and Aβ-AlO, confirming the cytotoxic nature of these oligomers. In the case of Aβ-FeO, we observed that the cell viability was not significantly reduced at the same tested concentrations. Furthermore, the drop in cell viability was observed to be more significant in the cases of Aβ-ZnO and Aβ-CuO as compared to Aβ-AlO, thus suggesting the inherent differences in cytotoxicity between the different oligomers (Figure 3A). To further investigate the mechanism of cytotoxicity of these oligomers, we assessed the amount of ROS production in the cells upon the treatment with these oligomers (Figures 3B and S7). The generation of ROS is an indication of cellular dysfunction which is often associated with the general cellular damage caused by oligomers.^36^ Upon incubation in the presence of 5 μM oligomers, ROS production was observed in the case of Aβ-ZnO and Aβ-CuO (Figure 3B). In the case of Aβ-AlO and Aβ-FeO, ROS production was not significantly increased. These results suggest the increase of ROS production to be one of the mechanisms of toxicity of these oligomers, as the more toxic Aβ-ZnO and Aβ-CuO also induced more ROS production than the other less toxic oligomers Aβ-AlO and Aβ-FeO.

We also assessed the ability of these oligomers in inducing Ca^2+^ ion influx, a phenomenon associated with the ability of the oligomers to permeabilize the lipid memebranes^34,37^ (Figures 3C and S7B). We observed an increase in the Fluo-4 AM fluorescence for cells incubated in the presence of Aβ-ZnO, Aβ-CuO, and Aβ-AlO, which was not apparent for Aβ-FeO at the same concentration. These results indicate that Aβ-ZnO, Aβ-CuO, and Aβ-AlO, but not Aβ-FeO, were able to induce a significant Ca^2+^ influx in the cells. Additionally, among the three active oligomers, we observed that Aβ-CuO induced the most Ca^2+^ influx, followed by Aβ-ZnO and Aβ-AlO. Taken together, the *in cell* measurements reveal the different relative toxicity and mechanism of cytotoxicity of the oligomers. Aβ-ZnO and Aβ-CuO are evidently toxic through inducing significant ROS production and Ca^2+^ influx, resultantly reducing cell viability when measured through means of MTT. Aβ-AlO appears to mainly induce Ca^2+^ influx without significantly inducing ROS production, which could explain its less potent toxicity in reducing cell viability. Lastly, Aβ-FeO appeared to be relatively nontoxic, as it did not appear to trigger any cytotoxic response in the different cellular assays at the concentrations tested.

### The Cytotoxicity of the Aβ Oligomers Is Correlated with Their Size

**Figure 4:**
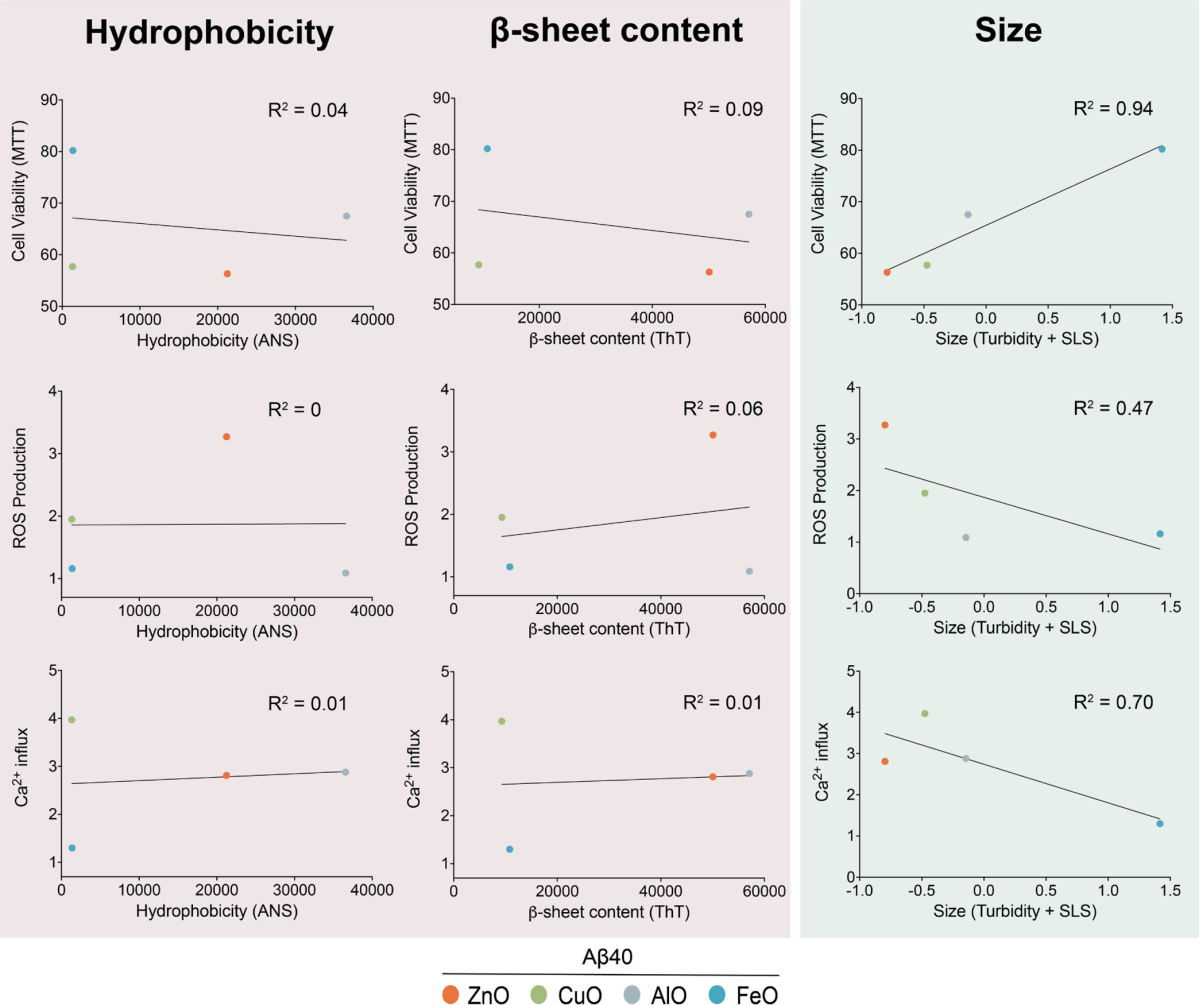
Structure–toxicity relationship of the four Aβ40 oligomer species studied in the work. Loss in cell viability (determined by MTT), increase in ROS production, and increase in Ca^2+^ influx in the presence of 5 μM of Aβ-ZnO (orange), Aβ-CuO (green), Aβ-AlO (gray), and Aβ-FeO (blue) as a function of their hydrophobicity (determined by ANS), their β-sheet content (determined by ThT), and their size (determined by turbidity and SLS). Solid black lines represent the linear regression between the data points. Note the strong correlation between the size of the oligomers and the cellular dysfunction markers.

The results obtained thus far have shown that the Aβ oligomers formed in the presence of different metal ions possess distinct physicochemical properties. Furthermore, *in cell* measurements have also shown that they exert different levels of cytotoxicity toward neuroblastoma cells. Thus, we sought to assess any relationship between the structural elements of these oligomers, with their ability to induce cellular dysfunction (Figures 4 and S8). These included the three structural properties: hydrophobicity (measured through ANS fluorescence), exposed β-sheet content (measured through ThT fluorescence), and size (measured through static light scattering and turbidity), and the three markers of cellular dysfunction: cell viability (measured through MTT), ROS production, and lipid membrane disruption (measured through Ca^2+^ influx-derived fluorescence). Assessment of the bivariate relationships shows that the two markers of cellular dysfunction (cell viability and membrane disruption) showed strong levels of correlation with the bulk measurements associated with the size of the oligomers only (static light scattering and turbidity) (Figure 4). Conversely, neither the exposed β-sheet content nor hydrophobicity of the oligomers appeared to correlate with the cellular dysfunction markers. These results suggest a correlation between the size of the oligomer with its cytotoxicity. We can observe that smaller oligomers such as Aβ-ZnO and Aβ-CuO appeared to be more toxic than the bigger oligomers Aβ-AlO and Aβ-FeO. This correlation appeared to be weaker in the case of ROS production, which could be attributed to the case of Aβ-AlO. Aβ-AlO, in particular, was able to induce a loss in cell viability and membrane disruption, but did not appear to trigger a substantial amount of ROS production. Nonetheless, taken together, across the 3 physicochemical properties of the oligomers measured, markers of cellular dysfunction appear to correlate inversely with the size of the oligomers, where we observed that populations of smaller oligomers tend to be significantly more cytotoxic than populations of the larger ones.

## Conclusions

We have investigated the effects of different metal ions in the kinetic stabilization of distinct forms of Aβ oligomers. These Aβ oligomers differ in their physicochemical properties and generate different levels of cellular dysfunction. Regression analysis has shown a correlation between the size of these oligomers and their cytotoxicity, suggesting that the size is likely to be a key structural determinant in the structure–toxicity relationship of Aβ oligomers. This finding supports previous reports of Aβ oligomers ex vivo,^38,39^ thereby illustrating the relevance of using these kinetically trapped oligomers as model systems in future studies pertaining to the molecular origins of Aβ oligomers in the pathology of AD. Further studies should also unravel the molecular origins of ion–Aβ interactions, such as the mechanisms and kinetics of metal binding to Aβ, and their possible roles in physiological ion homeostasis. This work has presented some examples of the myriad oligomers that can be generated in the presence of different cellular components, indicating that therapeutic strategies aimed at targeting the formation of Aβ oligomers should take into account their diversity in structure and cytotoxic effects.

## Materials and Methods

### Preparation of Compounds and Chemicals

ZnCl_2_, CuCl_2_, AlCl_3_, and FeCl_2_ were purchased in anhydrous forms from Sigma-Aldrich, and the salts were dissolved as 10 mM stocks in Milli-Q water and the solutions filtered. All chemicals used were purchased at the highest purity available.

### Preparation of Aβ Peptides

The recombinant Aβ(M1–40) peptide (MDAEFRHDSGY EVHHQKLVFF AEDVGSNKGA IIGLMVGGVV), here called Aβ40, was expressed in the *Escherichia coli* BL21 Gold (DE3) strain (Stratagene, CA) and purified as described previously with slight modifications.^40−42^ Briefly, the purification procedure involved sonication of *E. coli* cells, dissolution of inclusion bodies in 8 M urea, ion exchange in batch mode on diethylaminoethyl cellulose resin, and lyophilization. The lyophilized fractions were further purified using Superdex 75 HR 26/60 column (GE Healthcare, Buckinghamshire, U.K.) in 50 mM ammonium acetate buffer, pH 8.5, and eluates were analyzed using SDS-PAGE for the presence of the desired protein product. The fractions containing the recombinant protein were combined, frozen using liquid nitrogen, and lyophilized.

### Kinetic Assays

Aβ40 was injected into a Superdex 75 10/300 GL column (GE Healthcare, Buckinghamshire, U.K.) at a flow rate of 0.5 mL/min and eluted in 20 mM Tris buffer (pH 7.4). The obtained monomer was diluted in a buffer to a desired concentration and supplemented with 20 μM ThT from a 2 mM stock. All samples were prepared in low-binding Eppendorf tubes and then pipetted into a 96-well half-area, black/clear flat-bottom polystyrene NBS microplate (Corning 3881), 80 μL/well, in the absence and presence of different molar equivalents of metal ions (ZnCl_2_, CuCl_2_, AlCl_3_, or FeCl_2_) or oligomers (Aβ-ZnO, Aβ-CuO, Aβ-AlO, or Aβ-FeO). The assay was then initiated by placing the microplate at 37 °C under quiescent conditions in a plate reader (FLUOstar Omega, BMGLabtech, Aylesbury, U.K.). The ThT fluorescence was measured through the bottom of the plate with a 440 nm excitation filter and a 480 nm emission filter.

### Preparation of Aβ40 Oligomers Stabilized by Metal Ions

To generate stable Aβ40 oligomers, 0.5 mg of the lyophilized Aβ40 peptide was dissolved in 300 μL of 100% HFIP (yielding 0.37 mM peptide) and incubated overnight at 4 °C, and the solvent was then allowed to evaporate under a gentle flow of N_2_. The peptide was then resuspended in DMSO at a concentration of 2.2 mM and sonicated twice using a bath sonicator for 10 min at room temperature. The peptide solution was then split into 4 identical aliquots and diluted in 20 mM Tris buffer, at pH 7.4, with 1 mM ZnCl_2_, CuCl_2_, AlCl_3_, or FeCl_2_, to a final concentration of Aβ40 of 100 μM, incubated at 20 °C for 20 h, and centrifuged at 21,000*g* for 15 min at 20 °C. The supernatant was discarded, and the pellet containing the oligomers was resuspended in 20 mM Tris buffer at pH 7.4. The concentration of the oligomers formed was determined by amino acid composition after acid hydrolysis and is given as monomer equivalents.

### Atomic Force Microscopy

High-resolution and phase-controlled AFM was performed on positively functionalized mica (TedPella, Inc.) substrates.^43^ The mica surface was cleaved and incubated for 1 min with 10 μL of 0.5% (v/v) (3-aminopropyl)triethoxysilane (APTES) from Sigma-Aldrich (St. Louis, MO), in Milli-Q water. Then, the substrate was rinsed three times with 1 mL of Milli-Q water and dried by a gentle stream of nitrogen gas. 5 μM samples containing Aβ-ZnO, Aβ-CuO, Aβ-AlO, or Aβ-FeO were then deposited onto the functionalized mica surfaces. The droplet was incubated for 10 min, then rinsed with 1 mL of Milli-Q water, and dried by a gentle stream of N_2_. The preparation was carried out at room temperature. AFM maps were realized by means of a JPK nanowizard2 system operating in tapping mode and equipped with a silicon tip (μmasch, 2 N m^–1^) with a nominal radius of 10 nm. Images were flattened by using the SPIP software (Image Metrology, Hørsholm, Denmark).

### Static Light Scattering

Static light scattering measurements of 50 μM Aβ-ZnO, Aβ-CuO, Aβ-AlO, or Aβ-FeO were performed with fixed parameters for the attenuator and cell position at 25 °C using the Zetasizer Nano-S instrument (Malvern). A low-volume (70 μL) disposable cuvette was used (BRAND, Wertheim, Germany).

### Turbidity Measurements

The absorbance of 40 μM Aβ-ZnO, Aβ-CuO, Aβ-AlO, or Aβ-FeO at 500 nm was measured using a plate reader (Clariostar, BMGLabtech) in spectral scan mode. Values at 500 nm were obtained after subtracting the signal from the buffer alone.

### Immuno-Diffusional Sizing Measurements

Microfluidic diffusional sizing was performed as previously reported.^44^ Microfluidic channels were fabricated by standard soft-lithography techniques using poly(dimethylsiloxane) (PDMS) on a master wafer, curing it at 65 °C for 3 h.^45^ The height of the channels was measured with a Bruker’s Dektak profilometer (Coventry, U.K.). In all cases, the length of the channel was 10 cm and the height 25 μm. A channel width of 80 μm was used. The flow in the channel was controlled by applying positive pressure at both inlets, buffer, and analyte, by using two syringe pumps (Cetoni neMESYS, Cetoni GmbH, Korbussen, Germany) at total flow rates in the range of 80–400 μL/h, being the analyte flow 19:40 of the total flow rate. The buffer and protein solutions were injected through a 1 mL syringe (HSW) connected with a plastic tubing (0.38 ID, 1.09 OD) into the PDMS device. Both fractions, 1 and 2, were collected by connecting the device into a low-binding tube with plastic tubing. The collection time varied between 30 min and 3 h, according to the sample volume needed and flow rate used. The BSA (0.1%) present in the buffer prevented the proteins from sticking to the PDMS channels. The samples were collected separately from both outlets of the device (diffused and non-diffused) and treated as indicated in the protocols of Cisbio Bioassays, Inc. (Codolet, France). The TR-FRET immunoassay (Human beta amyloid beta peptide 1-40 kit) readings were performed on a plate reader (Clariostar, BMGLabtech with emmision at 620 nm and 650 nm) in white polystyrene plates with volumes of 20 μL per well. The incubation time before reading was 90 min at RT. The labeled antibodies, as well as the proteins, were diluted in the 50 mM sodium phosphate buffer, pH 7.4, BSA (0.1%). Monomeric Aβ40 was used for the standard curve of the Aβ40 monomer experiment and the respective oligomer for the sizing of each of them, in serial 1:2 dilutions with a starting concentration of 2 nM. The diffused and non-diffused experimental ratio were further compared to the ratio obtained with particle-based simulation (basis functions) to determine the corresponding average hydrodynamic radius.

### ANS Binding Measurements

40 μM Aβ-ZnO, Aβ-CuO, Aβ-AlO, or Aβ-FeO were added to a solution of ANS in 20 mM Tris, pH 7.4 to obtain a 3-fold excess of dye (120 μM). The emission spectra (excitation at 380 nm) were recorded at 37 °C by using a plate reader (Clariostar, BMGLabtech)

### ThT Binding Measurements

6 μM Aβ-ZnO, Aβ-CuO, Aβ-AlO, or Aβ-FeO were added to a solution of ThT in 20 mM Tris, pH 7.4 to obtain a final ThT concentration of 20 μM. The emission spectra (excitation at 440 nm) were recorded at 37 °C using a plate reader (Clariostar, BMGLabtech). The stability of the oligomers was measured by diluting preformed oligomers to a final concentration of 10 μM in the presence of 20 μM ThT into 20 mM Tris, pH 7.4, and recorded at 37 °C for 18 h.

### Fourier Transform Infrared (FTIR) Spectroscopy

Aβ-ZnO, Aβ-CuO, Aβ-AlO, and Aβ-FeO samples were centrifuged at 21,000*g* for 15 min at 20 °C, and the pellets resuspended in 5–10 μL of 20 mM Tris, pH 7.4 buffer to achieve a final protein concentration of 2.8 mM (monomer equivalents). Fourier transform infrared spectroscopy was performed using a Bruker Vertex 70 spectrometer equipped with a diamond ATR element (Bruker, Billerica, MA). Spectra were acquired with a resolution of 4 cm^–1^ and processed by means of the Bruker software. For each sample, 2 spectra were averaged (each spectrum obtained from 128 scans), and then the second derivative was calculated applying a Savitzky-Golay filter (second order, 12 points).

### Dot Blot Assay

2 μL aliquots of 5 μM Aβ-ZnO, Aβ-CuO, Aβ-AlO, and Aβ-FeO samples were spotted on a nitrocellulose membrane with a pore size of 0.2 μM. The membranes were blocked for 1 h (PBS, 0.1% (v/v) Tween, 5% (v/v) skimmed milk) and incubated overnight with 1:1000 6E10 (Biolegend, San Diego, CA), OC (Merck, Darmstadt, Germany), or A11 (Invitrogen, Carlsbad, CA) primary antibody diluted in blocking solution. They were then incubated for 1 h with Alexa488-conjugated secondary antibodies diluted in blocking solution at 1:5000. Membranes were washed in PBS, 0.1% Tween, between each incubation step. Fluorescence was detected by using a ChemiDoc Imager (Bio-Rad, Hercules, CA).

### Cell Cultures

Human SH-SY5Y neuroblastoma cells (A.T.C.C., Manassas, VA) were cultured in Dulbecco’s modified Eagle’s medium (DMEM)-F12+GlutaMax supplement (Thermo Fisher Scientific, Waltham, MA) with 10% fetal bovine serum. The cell cultures were maintained in a 5.0% CO_2_ humidified atmosphere at 37 °C and grown until 80% confluence for a maximum of 20 passages.

### MTT Assay

SH-SY5Y cells were transferred into a 96-well plate and treated for 24 h at 37 °C in the absence or presence of different concentrations of Aβ-ZnO, Aβ-CuO, Aβ-AlO, or Aβ-FeO. Then, cell cultures were incubated with 0.5 mg/mL 3-(4,5-dimethylthiazol-2-yl)-2,5-diphenyltetrazolium bromide (MTT) solution at 37 °C for 4 h and subsequently with cell lysis buffer (20% SDS, 50% *N*,*N*-dimethylformamide, pH 4.7) at 37 °C for 3 h. Absorbance values of blue formazan were determined at 590 nm using a plate reader (Clariostar, BMGLabtech), and cell viability was expressed as the percentage of MTT reduction in treated cells compared to untreated cells (taken as 100%).

### ROS Production Assay

The ROS production was measured using the Fluorometric Intracellular ROS kit MAK143 (Sigma-Aldrich, St. Louis, MO) according to the manufacturer’s protocol. In brief, SH-SY5Y cells were seeded in black polystyrene 96-well plates for 24 h and then treated in the absence or in the presence of 5 μM Aβ40 oligomers. The ROS production was monitored over time by measuring the emission of fluorescence at 520 nm (excitation at 490 nm) at 37 °C using a plate reader (BMGLabtech, Aylesbury, U.K.).

### Ca^2+^ Influx Assay

The cytosolic Ca^2+^ levels were measured by exposing SH-SY5Y cells loaded with 2 μM Fluo-4 AM to Aβ40 oligomers. The emitted fluorescence was recorded after excitation at 488 nm by acquiring pictures every 10 min and quantified using the fluorescence microscope IncuCyte S3 Live Cell Analysis System (Essen Bioscence).

## Supporting Information Available

Kinetic profiles of Aβ40 aggregation in the presence of metal ions; IDS measurements of Aβ-O; dot blot assay of Aβ-O; stability measurements of Aβ-O; seeding ability of Aβ-O on Aβ40 aggregation; cell viability in Tris buffers with ions; kinetics of cell viability in the presence of Aβ-O; and correlation matrix plot of Aβ-O between physicochemical properties and cellular dysfunction markers (PDF)

## Abbreviations

AD: Alzheimer’s disease
Aβ: amyloid-β peptide
Aβ40: 40-residue form of Aβ;
