# Supplementary material for: A Relationship between the Structures and Neurotoxic Effects of Aβ Oligomers Stabilized by Different Metal Ions
Source: ACS Chem Neurosci. 2024 Feb 28;15(6):1125–34. doi: 10.1021/acschemneuro.3c00718 (PMC10958495; doi:10.1021/acschemneuro.3c00718)
Supplement: Supplementary file 1 — cn3c00718_si_001.pdf [file cn3c00718_si_001.pdf]

SUPPORTING INFORMATION

A Relationship Between the Structures and Neurotoxic Effects of Aβ Oligomers Stabilized by Different Metal Ions

Sean Chia[1,4], Rodrigo Lessa Cataldi[1], Francesco Simone Ruggeri[1,5], Ryan Limbocker[1,6], Itzel Condado-Morales[1], Katarina Pisani[1], Andrea Possenti[1], Sara Linse[2], Tuomas P. J. Knowles[1,3], Johnny Habchi[1], Benedetta Mannini[1,7+], Michele Vendruscolo[1+]

[1]*Centre for Misfolding Diseases, Yusuf Hamied Department of Chemistry, University of Cambridge, Cambridge CB2 1EW, UK*
[2]*Department of Biochemistry & Structural Biology, Center for Molecular Protein Science, Lund University, PO box 124, 221 00 Lund, Sweden*
[3]*Department of Physics, Cavendish Laboratory, Cambridge CB3 0HE, UK*
[4]*Present address: Bioprocessing Technology Institute, Agency of Science, Technology and Research (A*STAR), Singapore 138668*
[5]*Present address: Laboratory of Organic Chemistry and Laboratory of Physical Chemistry, Wageningen University & Research, Stippeneng 6708 WE, the Netherlands*
[6]*Present address: Department of Chemistry and Life Science, United States Military Academy, West Point, New York 10996, USA*
[7]*Present address: Department of Experimental and Clinical Biomedical Sciences, Section of Biochemistry, University of Florence, Florence 50134, Italy*

[+]Corresponding author: benedetta.mannini@unifi.it, mv245@cam.ac.uk

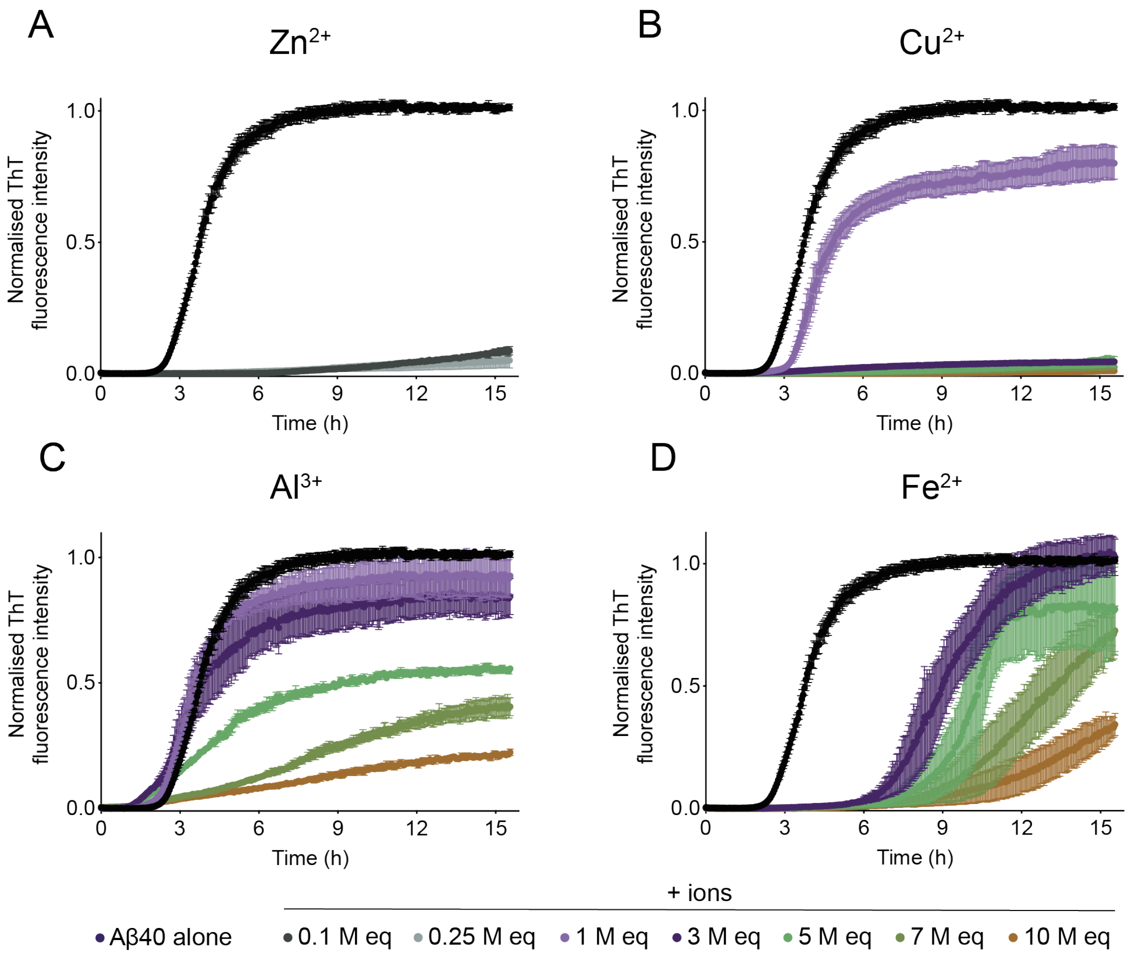

Figure S1. Metal ions inhibit the aggregation process of Aβ40. (**A-D**) Kinetic profiles of a 10 μM solution of Aβ40, either in the absence (black), or in the presence of increasing molar equivalents of either Zn^{2+} ions (**A**), Cu^{2+} ions (**B**), Al^{3+} ions (**C**), or Fe^{2+} ions (**D**). Error bars represent the s.e.m. (N=2).

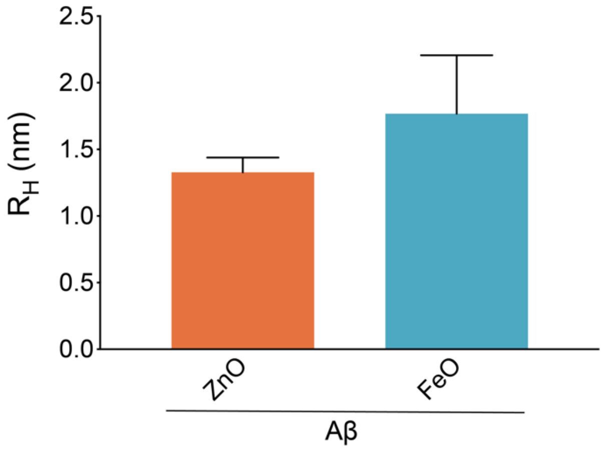

Figure S2. Size of Aβ40 oligomers stabilised by different metal ions determined by immune-diffusional sizing (IDS). Hydrodynamic radius of 2 nM Aβ-ZnO and Aβ-FeO in 20 mM Tris, pH 7.4 with 0.01% tween determined by IDS. Error bars represent the s.e.m. (N=2).

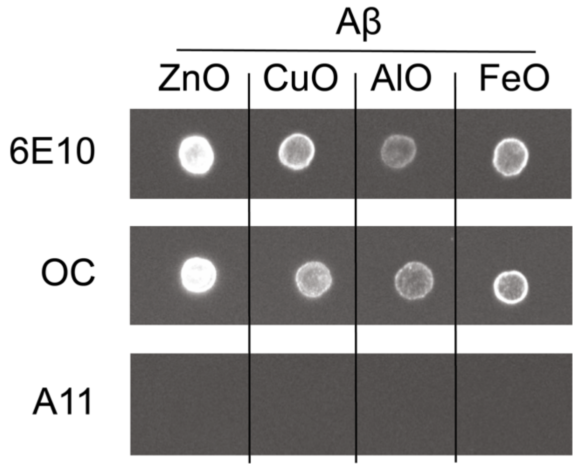

Figure S3. Dot blot assay of the four Aβ40 oligomer species in this work. Aβ-O samples were probed with the sequence-specific antibody 6E10, and the conformational specific antibodies OC and A11. A strong reactivity of the oligomers to the OC antibody is observed, while no significant signal is observed to the A11 antibody.

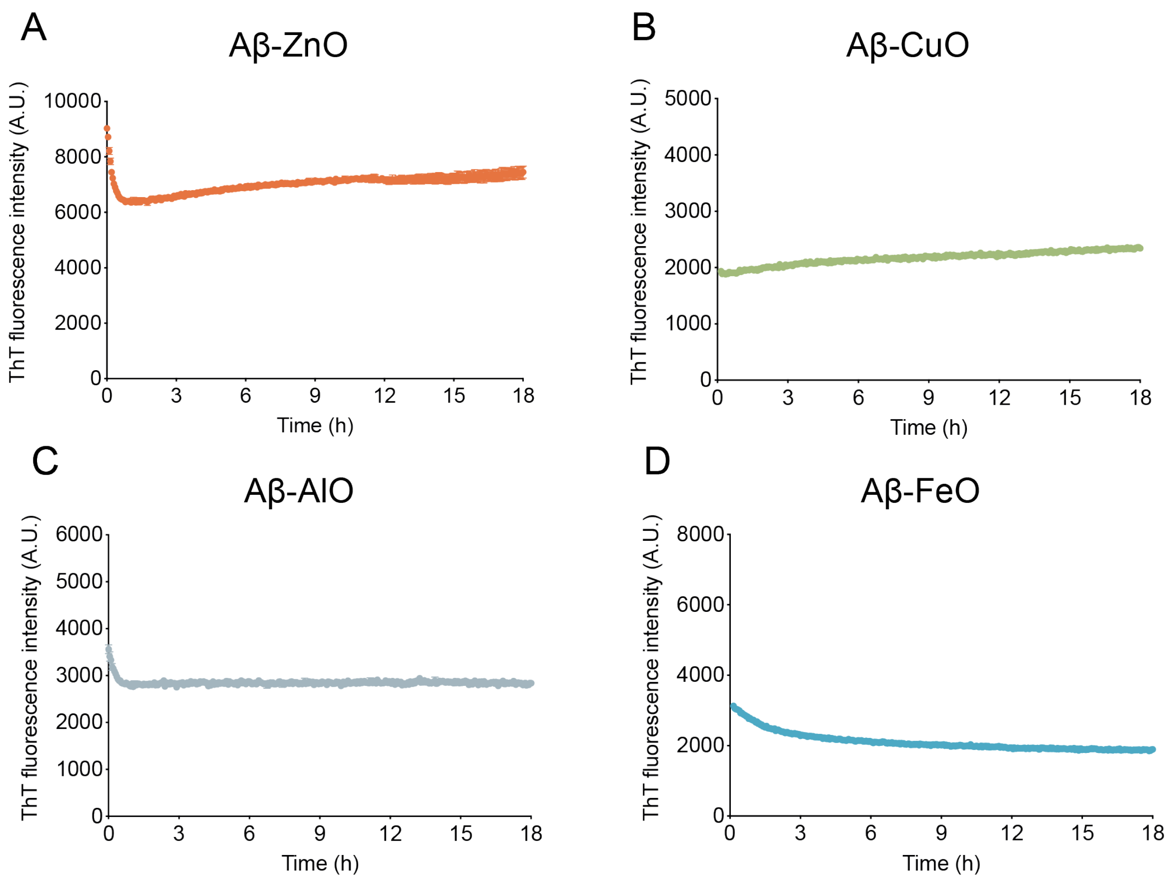

Figure S4. Stability of four Aβ40 oligomer species over time. (**A-D**) Kinetic profiles of a 10 μM solution of Aβ-ZnO (orange), Aβ-CuO (green), Aβ-AlO (grey) and Aβ-FeO (blue) over time at 37 °C measured through ThT fluorescence intensities. Error bars represent the s.e.m. (N=3).

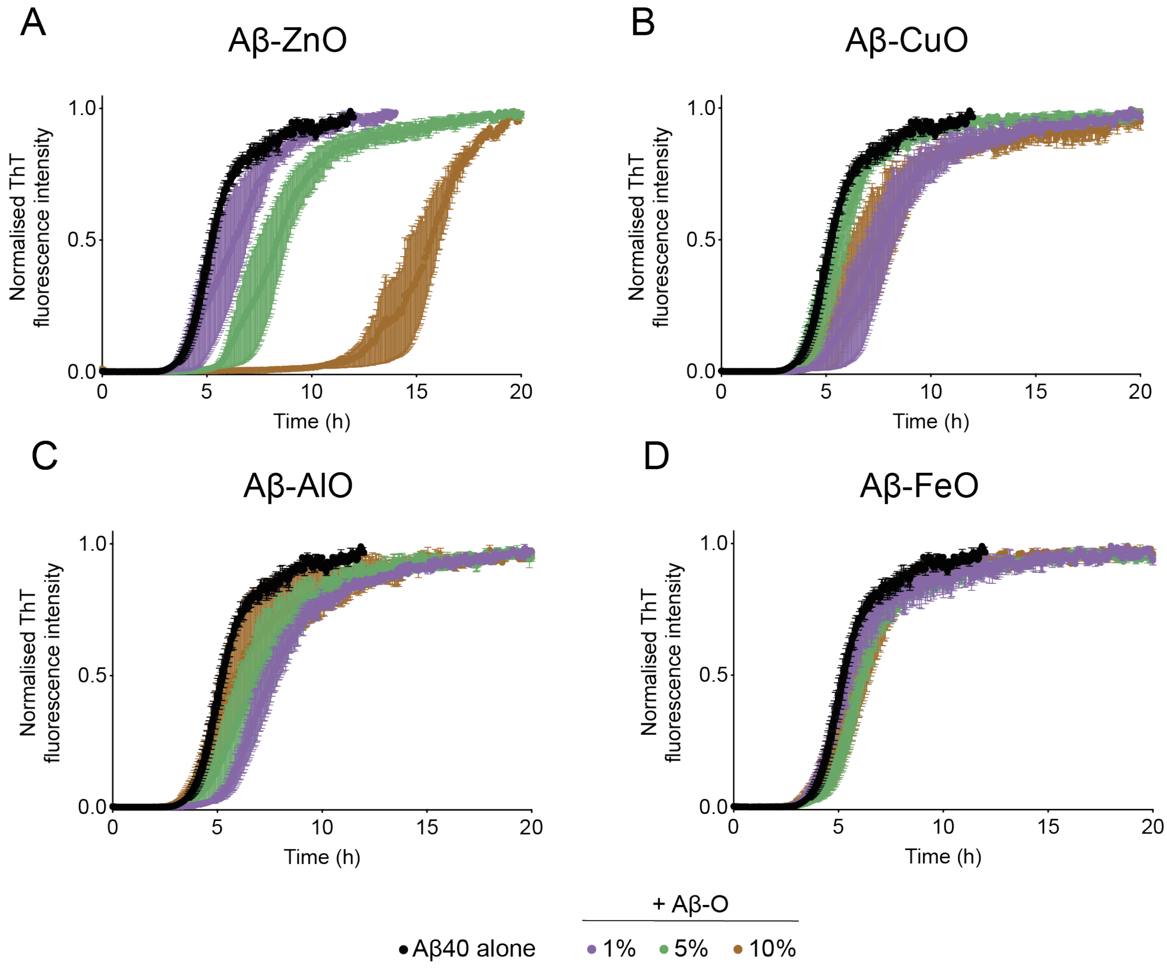

Figure S5. Seeding ability of Aβ40 oligomer species on the aggregation process of Aβ40. (**A-D**) Kinetic profiles of a 10 μM solution of Aβ40, either in the absence (black), or in the presence of increasing amounts of either Aβ-ZnO (**A**), Aβ-CuO (**B**), Aβ-AlO (**C**), or Aβ-FeO ions (**D**). Error bars represent the s.e.m. (N=3).

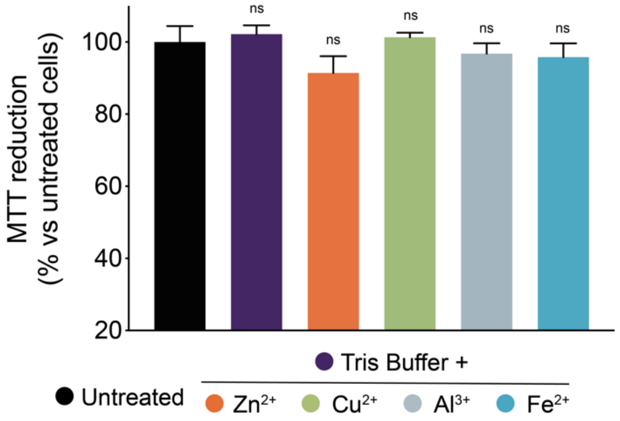

Figure S6. Cell viability in the presence of the four diluting Tris buffers used in this work. Viability of cells as determined by MTT reduction in the presence of 20 mM Tris pH 7.4, either alone, or in the presence of 1 mM of $ZnCl_2$, $CuCl_2$, $AlCl_3$, or $FeCl_2$ (represented in different colours). Error bars represent the s.e.m. (N=6).

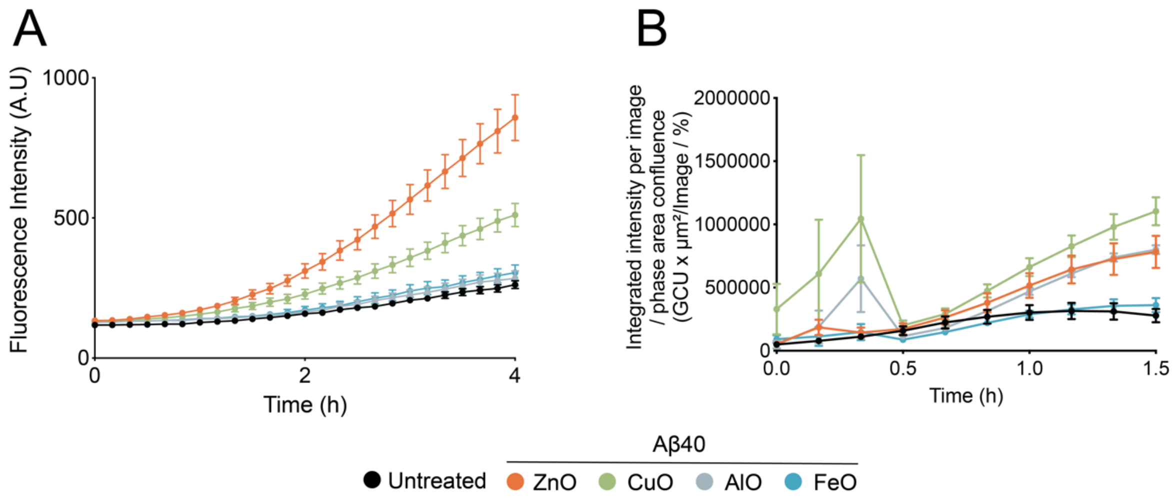

Figure S7. Measurement of cell viability over time in the presence of the four Aβ40 oligomer species studies in this work. **(A,B)** Kinetics at 37 °C of either ROS production (**A**) or Ca^{2+} influx (**B**) of cells in the absence, and presence of 5 μM of Aβ-ZnO (orange), Aβ-CuO (green), Aβ-AlO (grey) and Aβ-FeO (blue). The end point measurement (4 h for ROS production, 1.5 h for Ca^{2+} influx) was used to calculate F/F_0 ratios as shown in Figure 3. Error bars represent the s.e.m. (N=3).

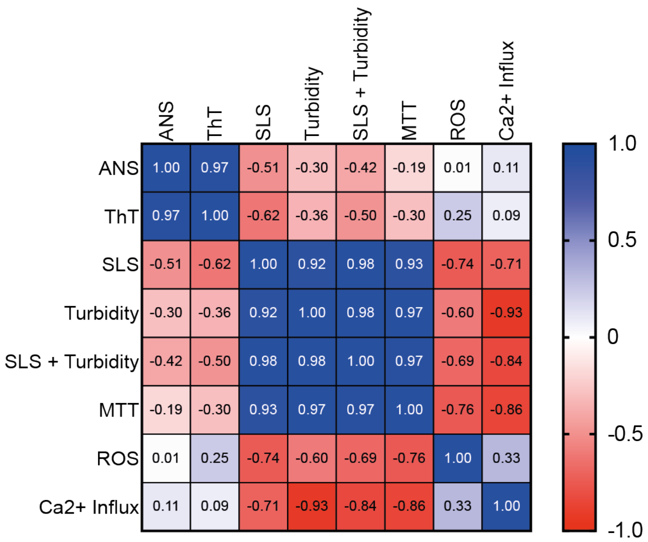

Figure S8. Correlation matrix plot between physico-chemical properties and cellular dysfunction markers. Heat map representing the Pearson's coefficient of correlation between the physico-chemical properties of Aβ-O and their corresponding cellular dysfunction readouts.
